# Domain and perception on community resilience: comparison between two countries

**DOI:** 10.3389/fpubh.2023.1157837

**Published:** 2023-07-17

**Authors:** Siska Nia Irasanti, Titik Respati, Ratna Januarita, Yuniarti Yuniarti, Hana Wei Jun Chen, Roy Rillera Marzo

**Affiliations:** ^1^Department of Public Health, Faculty of Medicine, Universitas Islam Bandung, Bandung, Indonesia; ^2^Department of Bussiness and Civil Law, Faculty of Law, Universitas Islam Bandung, Bandung, Indonesia; ^3^Department of Anatomy, Faculty of Medicine, Universitas Islam Bandung, Bandung, Indonesia; ^4^Department of Public Health, Management and Science University, Shah Alam, Malaysia

**Keywords:** community, resilience, COVID-19, domain, perception

## Abstract

The COVID-19 outbreak demonstrates how unprepared the world is for a different type of crisis, especially non-physical calamities. Revitalizing community involvement in disaster management is essential for making a community resilient. Due to differing sociocultural contexts, the resilience perceptions of communities in different parts of the world may vary. This study aims to understand community resilience factors after the COVID-19 disaster in Indonesia and Malaysia. Data from 2034 questionnaires using 5-interrelated domains in the Communities Advancing Resilience (CART) Toolkit Survey were collected. This study was conducted across Indonesia and Malaysia from March to April 2022. A quantitative-based cross-sectional study design and convenience sampling were applied. Respondents for this study were Indonesian and Malaysian citizens above 18 years of age who met the inclusion criteria. A total of 2034 respondents, 715 from Indonesia and 1,315 from Malaysia responded to the survey. The results suggest that Indonesian and Malaysian communities’ Transformative Potential and Informative-Communication domains differ significantly. Indonesian communities demonstrated a higher mean value on Transformative Potential domains than Malaysian communities did, while Malaysian communities indicated a higher mean value on Informative-Communication domains. This study concludes that compared to Malaysian communities, Indonesian communities have a more significant potential for transformation because they can frame collective experiences, gather, and analyze pertinent data, evaluate community performance, and develop resilience-building abilities. On the other hand, Malaysian communities are more resilient in providing information and communication. We found the need to develop a community resilience model that incorporates specific cultural and local requirements. Cooperation between the two countries would open many possibilities to emphasize the capability to bounce back sooner after a catastrophe such as the COVID-19 pandemic and achieve Sustainable Development Goals.

## 1. Introduction

The unique coronavirus (COVID-19) pandemic has been exerting pressure on the entire planet. It has demonstrated how unprepared the world is for non-physical disasters, a category of disasters that is frequently disregarded. It provides a unique learning opportunity because no one can predict with certainty when the COVID-19 pandemic will cease. It provides a unique learning opportunity because no one can predict with certainty when the COVID-19 pandemic will cease (1). People’s physical and emotional health is now seriously threatened by this pandemic, which has profoundly affected day-to-day living and psychological repercussions globally. At present, it is unimaginable how this pandemic will affect public health. More than 210 nations have been affected by the pandemic, and most are still undergoing various infection control procedures, such as lockdowns, quarantines, wearing masks, and social distancing. Most notably, the lockdowns decreased employment and incomes, which caused an increase in extreme poverty, Some international institutions stated that the Covid-19 pandemic disrupted the progress of achieving Sustainable Development Goals (SDGs). The COVID-19 pandemic has clarified that resilience-based approaches to poverty reduction and climate change adaptation are needed (2–4). To deal with shocks and increase resilience, it is urgently necessary to reassess the design and implementation of poverty reduction efforts for infrastructure and basic services, social safety systems, and health (5).

Another aspect that could influence future public health emergencies is global climate change. The World Economic Forum reported that climate change was linked to the world’s top risks in 2019 (6). Community involvement is important when managing disasters, as it helps make citizens more resilient during pandemics. Social networks can help people find information about a disaster before it occurs and assist them during and after the incident. Thus, social connectedness plays a vital role in health security. The government helps build community organizations that can help communities recover from disasters. These organizations help communities to be better prepared for disasters and to come back stronger afterward (7). The institutional response to any natural or artificial disaster begins at the local level, and it is here that preparation efforts are perhaps most critical (8).

Disaster is always related to emergencies. Public health faces a challenge because it needs to prepare limited resources and personnel to focus on pre-event preparations due to the range of possible emergencies. A focus on building, exercising, and sustaining public health capabilities are needed to effectively respond to most types of emergencies under an all-hazards approach. Disaster Risk Reduction (DRR) involves multiple disaster preparedness and response planning sectors. Wider sectors of society are needed to participate in planning for preventive or response activities related to particular threats in DRR planning. This approach was codified in the Sendai Protocol for Disaster Risk Reduction 2015–2030 (9). Resilience refers to developing the ability or capacity to build back better after a disaster (10). By using community resilience as a framework, we may better comprehend a community’s enduring ability to overcome and recover from hardship (11). A resilient community is constructed based on four attributes: strength, capital, temporal, and level of achievement. For example, a lack of flood resilience can mean that the impacts of floods do not go as planned and can undermine progress toward Sustainable Development Goals (SDGs). Therefore, to ensure that flood impacts do not cause the SDGs to be undermined, greater investments need to be made in DRR, climate change adaptation, and climate-smart development (12). Different cultures affect community resilience; for example central Eurasia has a vision of a better tomorrow, which stimulates the mobilization of inherent resources, communal support infrastructures, and the resolve needed to cope with the crisis (13). A study in 2021 concluded that in both Indonesia and Malaysia, the government plays a significant role in developing cooperatives and fostering the growth of the entity resilience (14).

Using community resilience as a framework can help comprehend a community’s enduring ability to overcome and recover from hardships. According to the conceptual framework developed by Chandra et al., some aspects of community resilience include communication, social connectivity, physical and psychological health, and integration and involvement of the organization. Civicmindedness and social duty are equally significant factors that this paradigm has not sufficiently highlighted. Combating COVID-19 requires teamwork and giving up personal preferences, especially when it comes to safeguarding vulnerable and at-risk populations (15).

This study assesses the community resilience level, SDGs related to community resilience, and the indicators for community resilience building. In accommodating the concern and interest in community resilience, a sound system that encompasses risk management and governance of community resilience will also be needed. Hence, this study provides initial mapping to support this objective (Table 1).

**Table 1:** Community resilience score (domain and perception).

Community resilience (domain and perception)	Malaysia	Indonesia	*p* value	Malaysia	Indonesia	*p* value
Statements	Mean (SD)	Mean (SD)		Mean (SD)	Mean (SD)	
*Domain 1: Connection and Caring*						
1. People in my community feel like they belong to the community.	0.77 (0.17)	0.68 (0.16)	**0.00**	0.78 (0.15)	0.77 (0.10)	0.05
2. People in my community are committed to the community’s well-being.	0.78 (0.17)	0.76 (0.13)	**0.04**			
3. People in my community have hope for the future.	0.77 (0.17)	0.81 (0.12)	**0.00**			
4. People in my community help each other.	0.80 (0.17)	0.80 (0.14)	0.78			
5. My community treats people fairly, regardless of their background.	0.78 (0.18)	0.79 (0.15)	0.61			
*Domain 2: Resources*						
6. My community supports programs for children and families	0.76 (0.19)	0.77 (0.13)	0.15	0.75 (0.017)	0.75 (0.11)	0.32
7. My community has the resources to take care of community problems.	0.73 (0.19)	0.74 (0.14)	**0.06**			
8. My community has effective leaders.	0.73 (0.20)	0.75 (0.14)	**0.01**			
9. People in my community can get the services they need.	0.75 (0.19)	0.75 (0.13)	0.87			
10. People in my community know where to go to get things done.	0.76 (0.18)	0.75 (0.12)	0.14			
*Domain 3: Transformative Potential*						
11. My community works with organizations and agencies outside the community to get things done.	0.72 (0.19)	0.74 (0.14)	**0.06**	0.74 (0.17)	0.76 (0.11)	0.00*
12. People in my community communicate with leaders who can help improve the community.	0.73 (0.19)	0.75 (0.13)	**0.07**			
13. People in my community work together on solutions to improve the community.	0.76 (0.18)	0.77 (0.13)	0.15			
14. My community looks at its successes and failures to learn from the past.	0.73 (0.19)	0.77 (0.13)	**0.00**			
15. My community develops skills and finds resources to solve its problems and reach its goals.	0.74 (0.19)	0.76 (0.12)	**0.00**			
16. My community has priorities and sets goals for the future.	0.73 (0.19)	0.77 (0.12)	**0.00**			
*Domain 4: Disaster Management*						
17. My community tries to prevent disasters.	0.74 (0.19)	0.75 (0.14)	0.11	0.73 (0.17)	0.74 (0.12)	0.20
18. My community actively prepares for future disasters.	0.72 (0.19)	0.73 (0.14)	0.49			
19. My community can provide emergency services during a disaster.	0.74 (0.18)	0.74 (0.14)	0.41			
20. My community has services and programs to help people after a disaster	0.73 (0.19)	0.74 (0.14)	0.15			
*Domain 5: Information and Communication*						
21. My community keeps people informed (for example, via television, radio, newspaper, internet, phone, and neighbors) about issues that are relevant to them.	0.75 (0.21)	0.73 (0.15)	**0.01**	0.75 (0.18)	0.72 (0.13)	0.00*
22. If a disaster occurs, my community provides information about what to do.	0.76 (0.19)	0.74 (0.14)	**0.04**			
23. I get information/communication through my community to help with my home and work life.	0.75 (0.19)	0.73 (0.15)	**0.01**			
24. People in my community trust public officials.	0.75 (0.19)	0.69 (0.16)	**0.00**			

The meaning of the bold values provided in table are the statistically significant values because its lower than 0.05.

## 2. Materials and methods

### 2.1. Study design, setting, and participants

This study was conducted across Indonesia and Malaysia from March to April 2022 A quantitative-based cross-sectional study design and convenience sampling were applied. The inclusion criteria for this study were that participants are residents aged 18 years and above and Indonesian and Malaysian citizens. All participants must be able to provide voluntary informed consent for this study. Google Forms was used to gather data, and the distribution method used word of mouth and emails. We also used web-based applications like Facebook, Twitter, Telegram, and WhatsApp.

The study sample size was calculated using the single population proportion formula, giving rise to the final sample size, *n* = 2034 (715 in Indonesia and 1319 in Malaysia). Non-probability convenience sampling was used for sample collection.

This study used an online questionnaire in Malay, English, and Bahasa Indonesia. Three experts performed the back-to-back translation to ensure the original meaning was preserved. The online questionnaire consisted of four sections: (1) Socio-demographic information; (2) Disaster experience, participation, training, active membership, and interest in deployment; (3) Communication and interaction between respondents and the community; and (4) Core community resilience. The remote data collection method was used to gather the data. Both descriptive and inferential statistics were used to analyze the data.

### 2.2. Study instrument

This study used the Communities Advancing Resilience Toolkit (CART) study. The theory and evidence-based CART research utilized in this application include 24 core community resilience elements to address five interconnected CART areas that reflect and contribute to community resilience. The following topics are covered in the current online CART tool manual that are (1) Connection and compassion assessed using questions about participation, shared values, support and compassion, justice, hope, and community diversity. (2) resources assessed using questions regarding the community’s natural, physical, human, social, and financial resources. (3) Transformative potential which is derived from the community’s capacity to articulate the collective experience, gather and evaluate relevant data, evaluate community performance, and build capacity; (4) disaster management, which taking into account activities for community prevention and mitigation, preparation, response, and recovery and the last (5) communication and information which assessed the sources of information and how communication conducted in the community during a disaster.

### 2.3. Data analysis

Participants were required to respond to each survey item on a scale of 1 to 6, ranging from “strongly disagree” to “strongly agree.” Average scores were calculated for each of the 24 individual core elements, each of the 5 CART domains, and the overall community resilience score. Regression models were used to assess covariates and associations with each domain score, overall community resilience score, community resilience strength, and community resilience challenge. Age, gender, marital status, employment status, experience of emergency/crisis while living in the community, and involvement were included as covariates. A stepwise procedure was used to select important covariates. Both descriptive and inferential statistics will be used to analyze data. Statistical analyses will be performed using Statistical Package for Social Sciences (SPSS) statistical software version 26.0. An Independent t-test will be performed, and a value of *p* < 0.05 will be considered statistically significant.

Data integrity will be maintained as questionnaires have been validated and tested for reliability before data collection. Participants provide valid phone numbers during the participation to ensure valid responses. The principal investigator will control Google form responses and be disabled for resubmission to prevent multiple responses from the same participant. Data collected will be stored carefully in Excel and is only accessible to the instigators conducting the study. Data encryption will be done to protect data confidentiality.

## 3. Results

All answers from all domains were converted to the top category that could be chosen so that all questions had a maximum value of 1 and a minimum of 0. Distributional assumption scoring was based on the mean, median, standard deviation, skewness, and kurtosis difference. The t-test was used because the conclusion of all questions in every domain fulfills the normal distributional assumption value.

Domains 3 (Transformative Potential) and 5 (Informative and Communication) were the domains of resilience community domains between Indonesia and Malaysia that are significantly different, with a value of *p* <0.05. The mean value of Domain 3 was higher in Indonesia, but Domain 5 had a higher mean value in Malaysia.

In Domain 1 (Connection and Caring), the mean values of questions 1 to 3 differed significantly between Indonesia and Malaysia. In contrast, the mean values of questions 1 (People in my community feel like they belong to the community) and 2 (People in my neighborhood are committed to the community’s well-being) in Malaysia were higher than in Indonesia. Still, the opposite occurred in question 3 (People in my community have hope about the future), where the mean value of the question in Indonesia was higher than that in Malaysia.

The mean values of questions 2 (My community has the resources it needs to take care of community problems) and 3 (My community has influential leaders) in Domain 2 (Resources) were statistically different between Indonesia and Malaysia; it was higher in Indonesia. Almost all questions in Domain 3 (Transformative Potential) had significantly different mean scores between the two countries. The median value of many questions in Indonesia was higher than that in Malaysia.

The opposite occurred in questions from Domain 4 (Disaster Management), where all questions had almost the same mean value between the two countries. However, there was no statistically significant difference in the mean value. All questions in Domain 5 (Information and Communication) had different mean values between the two countries, where Malaysia’s mean value was greater than that of Indonesia.

The difference in the mean values was in Domain 1 (Connection and Caring), 3 (Transformative Potential), and 5 (Information and Communication). Malaysia had a higher mean value than Indonesia in Domain 1 (Connection and Caring) and 5 (Information and Communication), whereas Indonesia had a higher mean value in Domain 3 (Transformative Potential). There was no significant difference in the mean value in Domain 2 (Resources) and 4 (Disaster Management).

## 4. Discussion

Resilience is the ability to bounce back or cope successfully with stress, which can occur at individual and community levels. Community resilience is a dynamic and multifaceted phenomenon determining a community’s ability to withstand a disaster’s impact and functioning in its aftermath. The institutional response to any disaster, natural or artificial, begins at the local level, and it is here that preparation efforts are perhaps the most critical. Under the all-hazards approach, the focus is on building, exercising, and sustaining public health capabilities that will be important for an effective response to most, if not all, public health emergencies. In practice, public health preparedness approaches often include all-hazards and scenario-based planning (9).

Domains 3 (Transformative Potential) and 5 (Informative and Communication) were the perception domains of resilience community domains between Indonesia and Malaysia that differed significantly. The mean value of Domain 3 was higher in Indonesia, but Domain 5 had a higher mean value in Malaysia. Almost all questions in Domain 3 had significantly different mean scores between the two countries. The mean value of many questions in Indonesia was higher than that in Malaysia. In this study, Domain 3 (transformative potential) consisted of statements that communities work with organizations and institutions outside the community to get things done. For example, the community communicates with leaders who can contribute to community improvement and collaborate on community improvement solutions. Additionally, the community sees successes and failures, learns from the past, develops skills, finds resources to solve problems and achieve goals, prioritizes, and sets goals for the future.

The most appropriate public health intervention in the wake of mass trauma is guided by the principles of psychological first aid, including ensuring access to safe housing and food and assisting people in reconnecting with family members and friends (16).

Given that *Gotong Royong* is an enduring cultural value in Indonesia, it is not a recent idea to refer to as Indonesia’s national identity. In addition, it has been suggested that *Gotong Royong* was profoundly ingrained in Indonesian society as a heritage of Indonesian ancestors and an intangible asset. People are urged to be helpful to one another in *Gotong Royong* so that everyone can maximize their capacity for personal growth and social interaction. From this viewpoint, *Gotong Royong’s* labor energy becomes essential for social solidarity, humanism, and unity (17).

Four ideas make up *Gotong Royong’s* cultural values: (1) people are a part of the community; (2) people depend in all facets on their fellow humans; (3) people must constantly uphold good relations with one another; and (4) people must be fair to one another (18). Furthermore, as a cultural value, *Gotong Royong* emphasizes working hard together by showing care toward each other to support collectivism, collaboration, and cooperation (19). Given that most Indonesians traditionally value their relationships with their neighbors and families, *Gotong Royong* as a cultural value cannot be separated from the activities of Indonesians’ daily lives (20).

Active participation can be in the form of support in material, financial, physical, mental, or spiritual skills, constructive thoughts or advice, or only praying to God (21, 22). Being aware of belonging to a powerful group leads to active participation in the *Gotong Royong* process (23). *Gotong Royong*, once put into practice, can serve as social capital for the neighborhood as it engages in the socioeconomic activity. Together, it will bring about favorable changes in people’s life (24).

There are elements of reciprocity, giving, and receiving in *Gotong Royong.* Everyone desires to assist others genuinely without seeking recognition or material gain. Consequently, this practice has the potential to significantly impact society, particularly in terms of commitment and social engagement (25). Completing tasks involving shared interests suggests that *Gotong Royong* is active (26). The constants in *Gotong Royong* are family, harmony, and assistance. The value of helping one another still exists in isolated rural areas. If there is a change in the value, it is slower than it would be in a village close to the city (27).

The internet and social media penetration has helped Malaysia stay abreast with other developed countries and impacted its civil society. Additionally, an increasing number of Malaysians reveal portions of their lives online, if not the whole. Individual media outlets and social networking platforms allow Malaysians to conduct transactions, gather information, and create and share ideas, information, and life stories (28). Without giving up, some local businesses began to take inventive actions by running extra promos, utilizing internet delivery, selling on Livestream, and running numerous other social media marketing (29). Furthermore, in March 2021, Malaysia had more than 27 million Facebook users, representing more than 80% of the country’s total population. This shows a growing need for mobile community networks, particularly during pandemics and lockdowns. Malaysians, especially those in the 16–29 age group, spend 9.17 h daily online and 3.01 h on social media (30).

## 5. Conclusion

The study findings revealed differences in community resilience between Malaysia and Indonesia. Malaysia appeared to have a more advanced level regarding the community’s connection and caring as well as effective information delivery and communication compared to Indonesia. Although there were minor differences in the mean value from the Transformative Potential aspect of the people, Indonesia score higher than Malaysia. In terms of community resources, both countries exhibit similar level. These findings showed that each country could learn and implement lessons to enhance each other community resilience. A strong community resilience positively impacts the overall well-being of the community. Collaborative effort between the two countries could unlock numerous possibilities to highlight the ability to recover after a catastrophe, such as the COVID-19 pandemic, and achieve Sustainable Development Goals (SDGs).

## 6. Recommendation and future research

We recommend further research on the need to develop a community resilience model that incorporates specific cultural and local requirements to validate the findings presented in this paper, especially to promote communities’ preparedness for a disaster with a leader’s involvement.

## Data availability statement

The original contributions presented in the study are included in the article/supplementary material, further inquiries can be directed to the corresponding author.

## Ethics statement

Written informed consent was obtained from the individual(s) for the publication of any potentially identifiable images or data included in this article.

## Author contributions

SI and RM: conceptualization. RM: methodology. TR: formal analysis. RJ: validation. TR and RJ: funding acquisition. HC: supervision. SI: writing –original draft. All authors have read and agreed to the published version of the manuscript.

## Funding

This research was funded by LPPM Universitas Islam Bandung, grant number 061/B.04/S.K./Rek/III/2022″ and “Universitas Islam Bandung funded the A.P.C.”

## Conflict of interest

The authors declare that the research was conducted in the absence of any commercial or financial relationships that could be construed as a potential conflict of interest.

## Publisher’s note

All claims expressed in this article are solely those of the authors and do not necessarily represent those of their affiliated organizations, or those of the publisher, the editors and the reviewers. Any product that may be evaluated in this article, or claim that may be made by its manufacturer, is not guaranteed or endorsed by the publisher.

## References

[1] Rahmaniah SE Syarmiati PRR. Community resilience and digital literacy model during the COVID-19 outbreak in Indonesia. BIRCI J. (2021) 4:12643–55. doi: 10.33258/birci.v4i4.3325

[2] WHO. (2022a). UN report finds COVID-19 is reversing decades of progress on poverty, healthcare and education, by activity assessed May 23rd 2023. Available at: https://www.un.org/uk/desa/un-report-finds-covid-19-reversing-decades-progress-poverty-healthcare-and (Accessed May 23, 2023).

[3] WHO. (2022b). The impact of COVID-19 on the welfare of households with children: an overview based on high frequency phone surveys, by activity assessed May 24th 2023. Available at: https://www.unicef.org/reports/impact-covid-19-welfare-households-children (Accessed May 24, 2023).

[4] WHO. (2022c). COVID-19 pushed 4.7 million more people in Southeast Asia into extreme poverty in 2021, but countries are well positioned to bounce Back — ADB, by activity assessed May 23rd 2023. Available at: https://www.adb.org/news/covid-19-pushed-4-7-million-more-people-southeast-asia-extreme-poverty-2021-countries-are-well (Accessed May 23, 2023).

[5] Muhyiddin NH. Indonesia development update a year of Covid-19: a long road to recovery and acceleration of Indonesia's development. Indonesian J Dev Plan. (2021) 5:1–19. doi: 10.36574/jpp.v5i1.181

[6] World Economic Forum. The global risks report. Cologny: World Economic Forum (2019).

[7] UNICEF. Indonesia Covid-19 response situation report. New York: UNICEF (2021).

[8] Garrett LC Magruder C Molgard CA. Taking the terror out of bioterrorism: planning for a bioterrorist event from a local perspective. J Public Health Manag Pract. (2000) 6:1–7. doi: 10.1097/00124784-200006040-00003, PMID: 10977608

[9] Prasher JM Redd SC. Preparing for and responding to public health emergencies. 16th ed. New York: McGraw Hill (2022).

[10] Mallick B Akter S. The poverty-vulnerability-resilience nexus: evidence from Bangladesh. Ecol Econ. (2013) 96:114–24. doi: 10.1016/j.ecolecon.2013.10.008

[11] Yip W Ge L Ho AHY Heng BH Tan WS. Building community resilience beyond COVID-19: the Singapore way. Lancet Reg Health West Pacific. (2021) 7:100091–9. doi: 10.1016/j.lanwpc.2020.100091 PMC7825823 33521745

[12] McDowell A Streed CG. Health disparities In: Keuroghlian AS Potter J Reisner SL, editors. Transgender and Gender Diverse Health Care. New York: The Fenway Guide, McGraw Hill (2022)

[13] Yuhertiana I Zakaria M Suhartini D Sukiswo HW. Cooperative resilience during the pandemic: Indonesia and Malaysia evidence. Sustainability. (2022) 14:5839. doi: 10.3390/su14105839

[14] Korosteleva EA Petrova I. What makes communities resilient in times of complexity and change? Camb Rev Int Aff. (2022) 35:137–7. doi: 10.1080/09557571.2021.2024145

[15] Azimi MA Syed Zakaria SA Majid TA Disaster risks from an economic perspective: Malaysian scenario. In: I.O.P. Conference Series: Earth and Environmental Science. Institute of Physics Publishing (2019).

[16] Rodriguez JM Vos F Sapir DG. Measuring psychological resilience to disasters: are evidence-based indicators an achievable goal? Environ Health. (2013) 12:1–10. doi: 10.1186/1476-069X-12-115 24359448 PMC3893382

[17] Hodriani Halking Ivana J. The culture of gotong royong of the multiethnic society in north Sumatera: how to introduce it to students through civic education? Adv Soc Sci Educ Human Res. (2018) 208:331–6. doi: 10.2991/icssis-18.2019.69

[18] Nur Bintari P Darmawan C. Peran pemuda sebagai penerus tradisi sambatan dalam rangka pembentukan karakter gotong royong. Jurnal pendidikan ilmu sosial. (2016) 25:57–76. doi: 10.17509/jpis.v25i1.3670

[19] Nisalia D. Aktualisasi nilai kekeluargaan (persaudaraan) dan nilai kegotongroyongan dalam permainan tarik tambang pada warga masyarakat RT 24 RW 06 Sidikan Umbulharjo Yogyakarta tahun 2012. Jurnal Citizenship: Media Publikasi Pendidikan Pancasila dan Kewarganegaraan (2012).

[20] Effendi TN. Budaya gotong royong masyarakat dalam perubahan sosial saat ini. Jurnal pemikiran sosiologi. (2013) 2:2–18. doi: 10.22146/jps.v2i1.23403

[21] Muqsith MA Pratomo RR Kuswanti A Muzykant VL. Social solidarity movement to prevent the spread of COVID-19 pandemic in Indonesia. Masyarakat, Kebudayaan dan Politik. (2021) 34:147–8. doi: 10.20473/mkp.V34I22021.147-158

[22] Khasanah N. Pengejewantahan nilai-nilai dalam pengembangan budaya gotong royong di era digital. Edukasi. (2013) 1:92–8.

[23] Rahayu S Ludigdo U Irianto G Nurkholis. Budgeting of school operational assistance fund based on the value of gotong royong. Soc Behav Sci. (2015) 211:364–9. doi: 10.1016/j.sbspro.2015.11.047

[24] Lukiyanto K Wijayaningtyas M. Gotong royong as social capital to overcome micro and small enterprise's capital difficulties. Heliyon. (2020) 6:e04879–8. doi: 10.1016/j.heliyon.2020.e04879 32984594 PMC7492849

[25] Basir PMI. Moral responsibility and wholeheartedness: a source of cohesion in Javanese society. Cosmop Civil Soc Interdisc J. (2021) 13:15–27. doi: 10.5130/ccs.v13.i1.7617

[26] Varensya F Hambali H Hariyanti H. Study the value of togetherness and gotong royong (team work) of flying duce race in limapuluh Kota regency. JED. (2022) 7:562–07. doi: 10.26618/jed.v7i3.7908

[27] Rosyani MF Napitupulu D Faust H. Gotong royong (cooperation) transformation of rural communities in Jambi Province, Indonesia. Jurnal Perspektif Pembiayaan dan Pembangunan Daerah. (2019) 7:103–07. doi: 10.22437/ppd.v7i1.7466

[28] Wok S Mohamed S. Internet and social media in Malaysia: development, challenges, and potentials. Intech Open Access J. (2017) 4–5. doi: 10.5772/intechopen.68848

[29] Jian Y Sze CC Fern YS Wan CY. Malaysian young Consumers' purchase intention through social media platforms during global pandemic. Int J Entrepreneur Bus Creat Econ. (2022) 2:14–22. doi: 10.31098/ijebce.v2i1.733

[30] Statista. (2021) Average time spent using online media in Malaysia in Q3 2020, by activity assessed in July 20th 2021. Available at: https://www.statista.com/statistics/803614/daily-time-spent-using-online-media-by-activity-malaysia (Accessed July 20, 2021).

